# The complete chloroplast genome of *Cinnamomum pittosporoides* reveals its phylogenetic relationship in Lauraceae

**DOI:** 10.1080/23802359.2019.1669503

**Published:** 2019-09-25

**Authors:** Xiong-Li Zhou, Li-Qin Zhang, Liu Yang, Feng Huang, Yue-Hua Wang, Xing Huang, Gang Deng, Shi-Kang Shen

**Affiliations:** a School of Life Sciences, Yunnan University, Kunming, Yunnan, China;; b Environment and Plant Protection Institute, Chinese Academy of Tropical Agricultural Sciences, Haikou, China;; c School of Agriculture, Yunnan University, Kunming, Yunnan, China

**Keywords:** *Cinnamomum pittosporoides*, phylogenetic, chloroplast genome, systematic position

## Abstract

*Cinnamomum pittosporoides* is an important timber plant endemic to southwest of China. In the present study, we sequenced the complete chloroplast genome of *C. pittosporoides* and used the data to reveal the species phylogenetic in Lauraceae. The complete chloroplast genome showed a circular genome of 152,730 bp size with 39.2% GC content. The genome is of typical structure and contains a pair of inverted repeat (IR) regions with 20,074 bp, separated by one large single-copy (LSC) with 93,722 bp and one small single-copy (SSC) regions with 18,860 bp. The genome contained 116 genes, including 82 protein-coding genes, 30 tRNA genes, and 4 rRNA genes. A phylogenetic tree reconstructed based on 26 chloroplast genomes reveals that *C. pittosporoides* is most related with *C. chago* in Lauraceae.

The genus *Cinnamomum* is an important part of tropical and subtropical evergreen broad-leaved forests with highly economical and ecological values (Wu et al. 2010). *Cinnamomum* consists of approximately 350 species distributed in the tropical and subtropical Asia, Australia, and the Pacific Islands, to tropical America and Africa (Li et al. 1981). Many *Cinnamomum* species are important materials for furniture, industry, traditional medicine, and condiments. *C Cinnamomum pittosporoides* is an important timber plant endemic to China. However, the phylogenetic relationship and systematic position of *C. pittosporoides* remain ambiguous. Herein, we sequenced and analyzed the complete chloroplast genome of *C. pittosporoides* using next-generation sequencing technology. Our aim was to retrieve chloroplast genome structure and reveal the species’ systematic position in Lauraceae

Fresh leaves of *C. pittosporoides* were collected from Xinping county in Yunnan Province, China (E:101°50′41.45″, N:24°6′11.88″). The specimen is stored at Yunnan University Herbarium (HYN-WYH171012). Total genomic DNA was extracted using a modified cetyltrimethylammonium bromide (CTAB) method (Doyle 1987). The sequencing library was constructed and quantified and then the paired-end (PE) libraries were generated using Illumina HiSeq 2500 platform. The whole genome sequencing was conducted by Novogene (Tianjin, China). We assembled the short reads into contigs using SPAdes, connected all contigs with Bandage, and manually removed redundant contigs. We mapped reads to the genome to check, proofread, and patch and finally obtained cycle complete plastomes. The cp genome was annotated through DOGMA (Wyman et al. 2004) and the boundaries of start and stop codons and intron/exon were checked manually using Geneious version 8.1.4. We confirmed all tRNA genes using online tRNAscan-SE (Schattner et al. 2005). The final complete plastomes were deposited in GenBank with accession numbers MN047450.

The cp genome of *C. pittosporoides* is a circular molecule of 152,730 base pairs (bp) with a pair of inverted repeats (IR) of 20,074 bp, separated by a large (LSC, 93,722 bp) and a small single copy (SSC, 18,860 bp). The overall GC content of *C. pittosporoides* cp genome is 39.2% and the corresponding values in LSC, SSC, and IR regions are 38.0, 34.0, and 44.4%, respectively. The cp genomes were annotated with 116 genes, including 82 protein-coding genes, 30 tRNA genes, and 4 rRNA genes. In the cp genomes of *C. pittosporoides*, 12 genes contain introns structure, whereas the genes of clpP and ycf3 have two introns. In addition, the rps12 gene was trans-spliced with exon 1 in the LSC region and exons 2 and 3 in the IR region.

To reveal the systematic position of *C. pittosporoides* in the Lauraceae, we performed a phylogenomic analysis using the chloroplast genomes sequences of 26 species (*Magnolia grandiflor*a and *Drimys granadensis* as outgroup) in PAUP version 4.0a with 1000 bootstrap replicates (Swofford 2002). The phylogenetic tree indicated that *C. pittosporoides* has a closer relationship with *C. chago* than other species in Lauraceae with 100% bootstrap value (Figure 1). The complete chloroplast genome of *C. pittosporoides* provides valuable genomic resources that will be used for revealing the species’ phylogeny, exploring genetic variations and designing conservation strategy.

**Figure 1.**
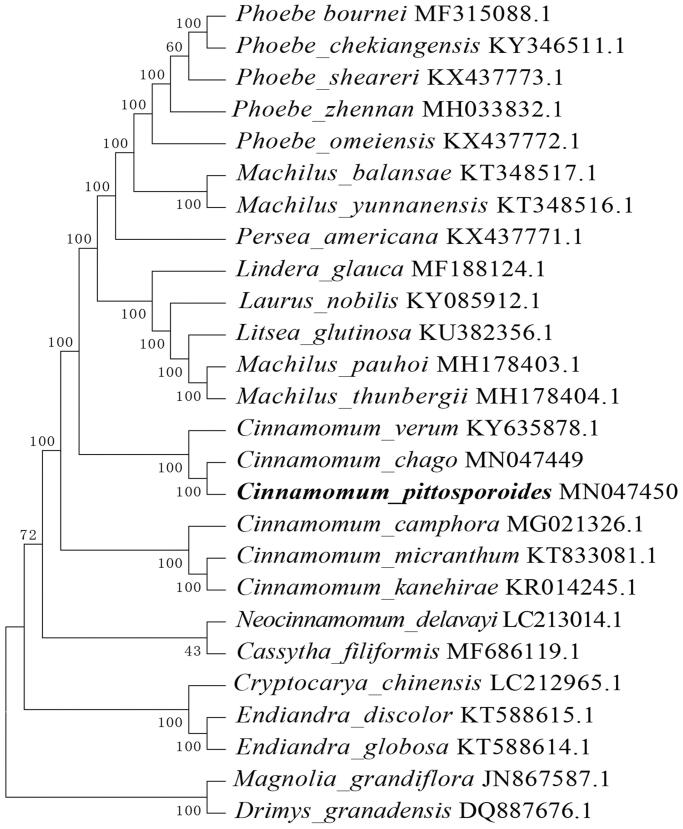
Phylogenetic position of *Cinnamomum pittosporoides* based on the complete chloroplast genome sequences of 26 species. Bootstraps were shown next to the node.
